# Transmission of Severe Acute Respiratory Syndrome Coronavirus 2 Among Residents and Employees in a Veterans Affairs Community Living Center: A 42-Month Prospective Cohort Study

**DOI:** 10.20411/pai.v9i1.691

**Published:** 2024-04-24

**Authors:** Chetan Jinadatha, Lucas D. Jones, Jennifer M. Hailes, Emma K. Marshall, Munok Hwang, Jennifer L. Cadnum, Hosoon Choi, Piyali Chatterjee, Ernest R. Chan, Peter A. Zimmerman, Nadim G. El Chakhtoura, Elie A. Saade, Curtis J. Donskey

**Affiliations:** 1 Medical Service, Central Texas Veterans Healthcare System, Temple, Texas; 2 School of Medicine, Texas A&M University, Bryan, Texas; 3 Department of Molecular Biology and Microbiology, Case Western Reserve University School of Medicine, Cleveland, Ohio; 4 Research Service, Louis Stokes Cleveland VA Medical Center, Cleveland Ohio; 5 Research Service, Central Texas Veterans Healthcare System, Temple, Texas; 6 Cleveland Institute for Computational Biology, Case Western Reserve University, Cleveland, Ohio; 7 The Center for Global Health & Diseases, Case Western Reserve University, Cleveland, Ohio; 8 Case Western Reserve University School of Medicine, Cleveland, Ohio; 9 Geriatric Research, Education, and Clinical Center, Louis Stokes Cleveland VA Medical Center, Cleveland, Ohio; 10 Division of Infectious Diseases and HIV Medicine, University Hospitals Cleveland Medical Center, Cleveland, Ohio

**Keywords:** SARS-CoV-2, COVID-19, nursing home, transmission, carbon dioxide, ventilation

## Abstract

**Background:**

Understanding routes of severe acute respiratory syndrome coronavirus 2 (SARS-CoV-2) transmission in long-term care facilities is essential for the development of effective control measures.

**Methods:**

Between March 1, 2020, and August 31, 2023, we identified coronavirus disease 2019 (COVID-19) cases among residents and employees in a Veterans Affairs community living center that conducted routine screening for asymptomatic COVID-19. Contact tracing was conducted to identify suspected transmission events, and whole genome sequencing was performed to determine the relatedness of SARS-CoV-2 samples.

**Results:**

During the 42-month study period, 269 cases of COVID-19 were diagnosed, including 199 employees and 70 residents. A total of 48 (24.1%) employees and 30 (42.9%) residents were asymptomatic. Sequencing analysis provided support for multiple events in which employees transmitted SARS-CoV-2 to co-workers and residents. There was 1 episode of likely transmission of SARS-CoV-2 from one resident to another resident, but no documented transmissions from residents to employees.

**Conclusions:**

Transmission of SARS-CoV-2 in the community living center predominantly involved transmission from employees to co-workers and residents. There is a need for improved measures to prevent transmission of SARS-CoV-2 by healthcare personnel.

## INTRODUCTION

Nursing home residents and employees have been substantially impacted by the coronavirus disease 2019 (COVID-19) pandemic [1]. Preventing severe acute respiratory syndrome coronavirus 2 (SARS-CoV-2) infections in nursing home residents is a priority because factors such as age, frailty, and comorbid illnesses place them at high risk for adverse outcomes [2]. Pre-symptomatic and asymptomatic individuals may contribute substantially to transmission in long-term care facilities [3–8]. Therefore, in addition to other control measures, serial testing of asymptomatic employees and residents has been recommended in outbreak settings [9]. Efforts to assess and remediate deficiencies in ventilation are also recommended as some COVID-19 outbreaks in nursing homes have been attributed in part to suboptimal ventilation [10–13].

Although transmission of SARS-CoV-2 among employees, residents, and visitors is often suspected in long-term care settings, the source of acquisition is often uncertain. Whole genome sequencing is a valuable tool that can complement contact tracing investigations of suspected transmission events [14]. However, the use of whole genome sequencing has been reported in relatively few investigations of SARS-CoV-2 transmission in nursing homes, and most were conducted during early stages of the pandemic [3, 6–9]. In these previous studies, sequencing provided evidence of SARS-CoV-2 transmission from employees and visitors in nursing homes to residents [3, 6–9]. Additional investigations that include sequencing are needed to examine current routes of transmission in nursing homes, including the potential for nursing home residents to transmit SARS-CoV-2 to other residents or staff. In the current study, we performed contact tracing and whole genome sequencing to investigate transmission of SARS-CoV-2 over a 42-month period in a Veterans Affairs community living center. Carbon dioxide monitoring was used to assess ventilation in the community living center.

## METHODS

### Setting

The VA Northeast Ohio Healthcare System includes a 215-bed acute-care hospital and an adjacent 174-bed community living center. The community living center provides skilled nursing and rehabilitative care. Four of the 6 community living center units occupy 4 floors of a building connected to the main hospital [8]. During the study period, all community living center residents were tested for SARS-CoV-2 by nasopharyngeal swab reverse transcriptase polymerase chain reaction (RT-PCR) prior to admission; during their stay, residents were tested by antigen once or twice each week if asymptomatic and by RT-PCR if respiratory symptoms occurred or if they had a high-risk exposure to COVID-19. Residents testing positive for SARS-CoV-2 were transferred to the hospital for the duration of isolation. Visitation was restricted throughout the facility during the initial 2 years of the pandemic and then during outbreak periods on individual wards.

Employees working in the community living center were required to wear medical procedure masks while in the community living center unless alone in a workspace behind closed doors, and they were screened once or twice weekly for COVID-19 by antigen testing. Prior to March 2023, it was recommended that employees eat meals alone at their desks and not sit together during break periods. Employees were asked to report any respiratory symptoms, and if symptoms developed, they were tested by RT-PCR and excluded from work until the results were available.

### Contact Tracing Investigations

The study protocol was approved by the Cleveland VA Medical Center's Institutional Review Board with a waiver of informed consent. Between March 1, 2020, and August 31, 2023, we identified COVID-19 cases among residents and employees in the community living center. The Infection Control Department conducted contact tracing in accordance with Centers for Disease Control and Prevention (CDC) recommendations [15]. Higher-risk exposures were defined as 15 minutes or more of continuous or cumulative contact within 6 feet without wearing both a facemask and eye protection that occurred within 2 days before symptom onset through the time when the source individual met the criteria for discontinuation of transmission-based precautions [15]. Contacts within 6 feet but for less than 15 minutes or while wearing both a facemask and eye protection were classified as lower-risk exposures [14, 16]. Asymptomatic employees or residents were offered testing if they were identified as being at risk through contact tracing investigations. In clusters with 3 or more cases, surveillance nasopharyngeal swab testing was conducted for all residents and employees on a unit.

All available nasopharyngeal swab specimens with RT-PCR cycle threshold of 35 or less were included in the sequencing analysis. Infection control records were reviewed to identify clusters of 3 or more COVID-19 cases on each ward in which nosocomial transmission was suspected based on contact tracing. Given that many employees worked on more than 1 ward in the facility, we hypothesized that transmission events would not be isolated to individual wards. Therefore, we examined the relatedness of sequenced respiratory samples on different wards during the same period to assess for potential transmission events that were not initially suspected based on contact tracing.

### Sequencing

Sequencing was performed as previously described [17]. RNA was extracted from positive nasopharyngeal swab specimens with the QIAamp Viral RNA Mini Kit (Qiagen) according to the manufacturer's instructions. After cDNA synthesis libraries for SARS-CoV-2 genomic sequencing were prepared using the COVIDSeq assay (Illumina), and using the Artic V4 primers. NextSeq Mid Output reagent kit v2 (Illumina) was used for sequencing with a read length of 2 x 150 bp on an Illumina NextSeq550 (Illumina).

### Sequencing Data Analysis

The raw sequencing data FASTQ file was uploaded to the BaseSpace sequence hub and a consensus FASTA file was generated with SARS-CoV-2 reference sequence (NC_045512.2 SARS-CoV-2 Wuhan-Hu-1, complete genome) using the DRAGEN COVID lineage App (Illumina) with default parameter. Stringent filtering criteria were used including only specimens with a minimum coverage of 95% or higher and 1000x median coverage depth in the final analysis. The clades were determined using Nextclade^beta^ (Version: 0.14.2) (https://clades.nextstrain.org/). The lineage was determined using the Pangolin tool (https://cov-lineages.org/resources/pangolin.html).

The consensus FASTA files were downloaded and processed through Bionumerics 7.6 (Applied Maths) for cluster and single nucleotide polymorphism (SNP) analysis. The SARS-CoV-2 plugin tool was used to analyze the SARS-CoV-2 genomic sequences. Sequences were analyzed for SNP differences relative to the NCBI reference sequence for SARS-CoV-2 (NC_045512). Sequences with ≤2 SNP differences were considered related if they belonged to the same clade. Sequences with 3 to 4 SNP differences were deemed to be possibly related if contact tracing indicated a plausible transmission event, and the sequences were of the same clade designation [14]. Using the advanced clustering tools, a similarity matrix was calculated based on the similarity coefficient between the isolates. The results of the similarity matrix were then used as input data in the complete linkage clustering algorithm to generate dendrograms and calculate SNP differences. Only samples that met our stringent filtering criteria were used to generate the dendrograms.

### Carbon Dioxide Monitoring to Assess Adequacy of Ventilation

Carbon dioxide monitoring was performed as previously described using an IAQ-MAX COW Monitor and Data Logger (CO2Meter, Inc) [18]. At nursing stations on 2 wards and in a large physical therapy area used by all wards of the community living center, carbon dioxide levels were continuously monitored and recorded every minute over 12 hours from 7 AM to 5 PM. The peak number of people present in these locations during monitoring was recorded. Additional readings were obtained over 20-minute to 60-minute periods in resident rooms (N=3), hallways on 3 wards, a conference room, lunchroom, and the main elevators. Carbon dioxide levels were recorded every minute. The readings in the conference room were recorded during a routine educational conference with 12 attendees. The readings in the elevators were recorded during routine elevator operation. For the resident room assessments, only study staff were present in the rooms; 2 staff members remained in the rooms with the doors closed. Based on recommendations from the Centers for Disease Control and Prevention (CDC), carbon dioxide readings above 800 ppm were considered an indicator of suboptimal ventilation requiring intervention [18, 19]. When available, the expected air changes per hour (ACH) based on design specifications were recorded for the areas or rooms where carbon dioxide readings were taken.

## RESULTS

### Contact Tracing Investigations

**Figure 1.**
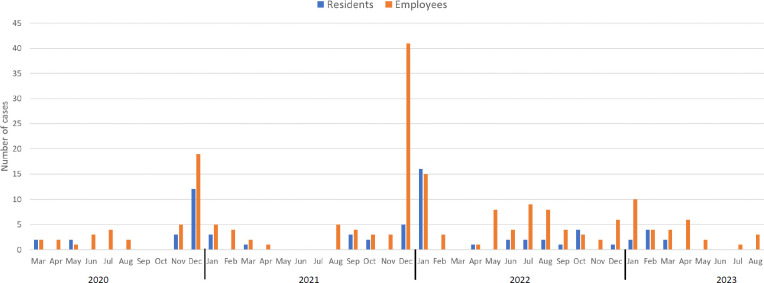
Timeline showing coronavirus disease 2019 (COVID-19) cases diagnosed in employees and residents in a community living center.

During the 42-month study period, 70 residents and 199 employees in the community living center were diagnosed with COVID-19 (Figure 1). Of the 199 employees, 104 (52.3%) were nurses, 46 (23.1%) were nursing assistants, 7 (3.5%) were physicians or physician's assistants, and 42 (21.1%) were ancillary staff. Of the 199 employees, 48 (24.1%) were asymptomatic and 151 (75.9%) were pre-symptomatic or symptomatic. In several cases, employees worked when they had mild respiratory symptoms that they initially attributed to other causes such as allergies, and subsequently were tested after their symptoms worsened. Of the 70 residents, 30 (42.9%) were asymptomatic and 40 (57.1%) were pre-symptomatic or symptomatic. Two residents died due to COVID-19 infection, and 2 additional residents had prolonged intensive care unit admissions for COVID-19. The peak in cases in the facility occurred from December 2021 through January 2022, coinciding with a surge of omicron variant BA.1.1 in Northeast Ohio.

During the study, the Infection Control Department investigated 15 ward-based clusters that involved 3 or more cases of COVID-19 (range, 3 to 13 cases) with suspected transmission among employees and/or residents. Eight of 15 (53.3%) clusters involved both residents and employees. In 6 of these clusters, transmission from infected employees to residents was suspected as employees on the same ward were diagnosed with COVID-19 within the 2 weeks prior to the resident cases; in 4 of these clusters, transmission from residents to employees was suspected as employees were exposed to infected residents prior to their COVID-19 diagnosis. Seven of the clusters only involved employees. Of the 15 clusters investigated, 4 were on unit 1, 3 were on unit 2, 4 were on unit 3 (ie, a dementia unit), 2 were on unit 4, and 2 were on unit 5. In 8 of the clusters, 1 or more infected employees worked on multiple units of the community living center.

### Sequencing Analysis

Of 143 samples submitted for sequencing, 72 (50.3%) with cycle threshold values of 35 or lower had minimum coverage of 95% and 1000x median coverage depth and were included in the analysis. Figure 2 provides dendrograms displaying the SNP differences between the viral sequences divided into periods during the pandemic when viruses with distinct lineages were detected, including B.1.2 (pre-delta), delta, and omicron lineages BA.1.1, BA.2, BA.4, BA.5/BE/BF, and BQ. Of the 15 ward-based clusters identified by contact tracing, 10 had 2 or more sequencing results associated with suspected transmission events available for analysis. Of these 10 clusters, 8 (80%) had sequencing results that provided evidence supporting SARS-CoV-2 transmission between 1 or more individuals.

Figure 2.**Dendrogram displaying the single nucleotide polymorphism differences between severe acute respiratory syndrome coronavirus 2 (SARS-CoV-2) viral sequences of residents and employees of a community living center diagnosed with coronavirus disease 2019 (COVID-19).** (A) B.1.2 lineage; (B) delta lineage; (C) BA.1.1 omicron lineage; (D) BA.2 omicron lineage; (E) BA.4 omicron lineage; (F) BA.5, BE, and BF omicron lineages; and (G) BQ omicron lineage. The Wuhan-Hu-1 reference genome is shown for comparison. The dates indicate the date of the positive test result. Ward numbers indicate wards 1 through 5. The labels shown in bold text in panels A and C indicate the employees and residents shown in Figure 3A and 3B, respectively. E, employee; R, resident.

**Figure 2A:**
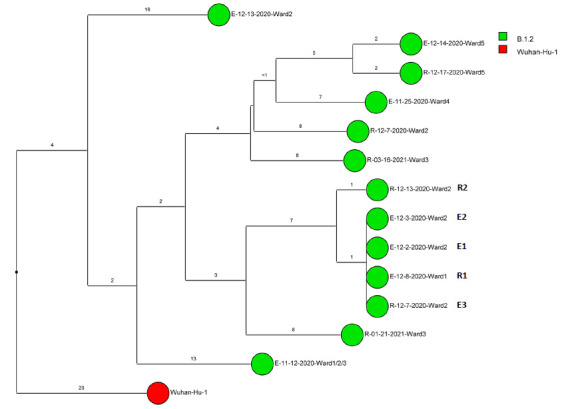
(B.1.2 [n=13])

**Figure 2B:**
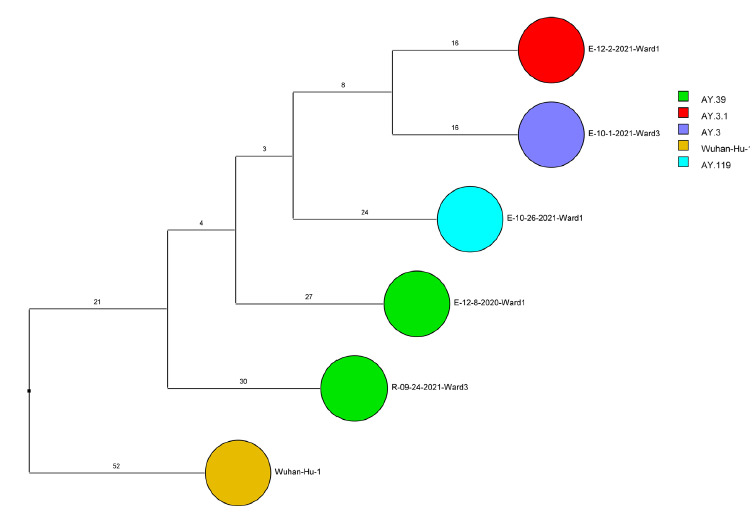
(Delta [n=5])

**Figure 2C:**
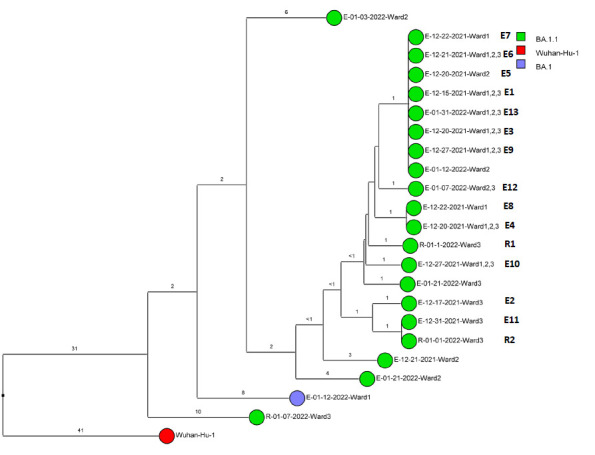
(Omicron BA.1.1 [n=22])

**Figure 2D:**
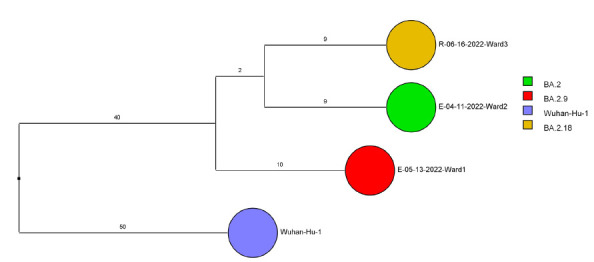
(Omicron BA.2 [n=3])

**Figure 2E:**
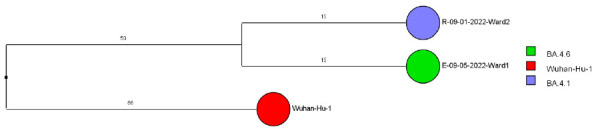
(Omicron BA.4 [n=2])

**Figure 2F:**
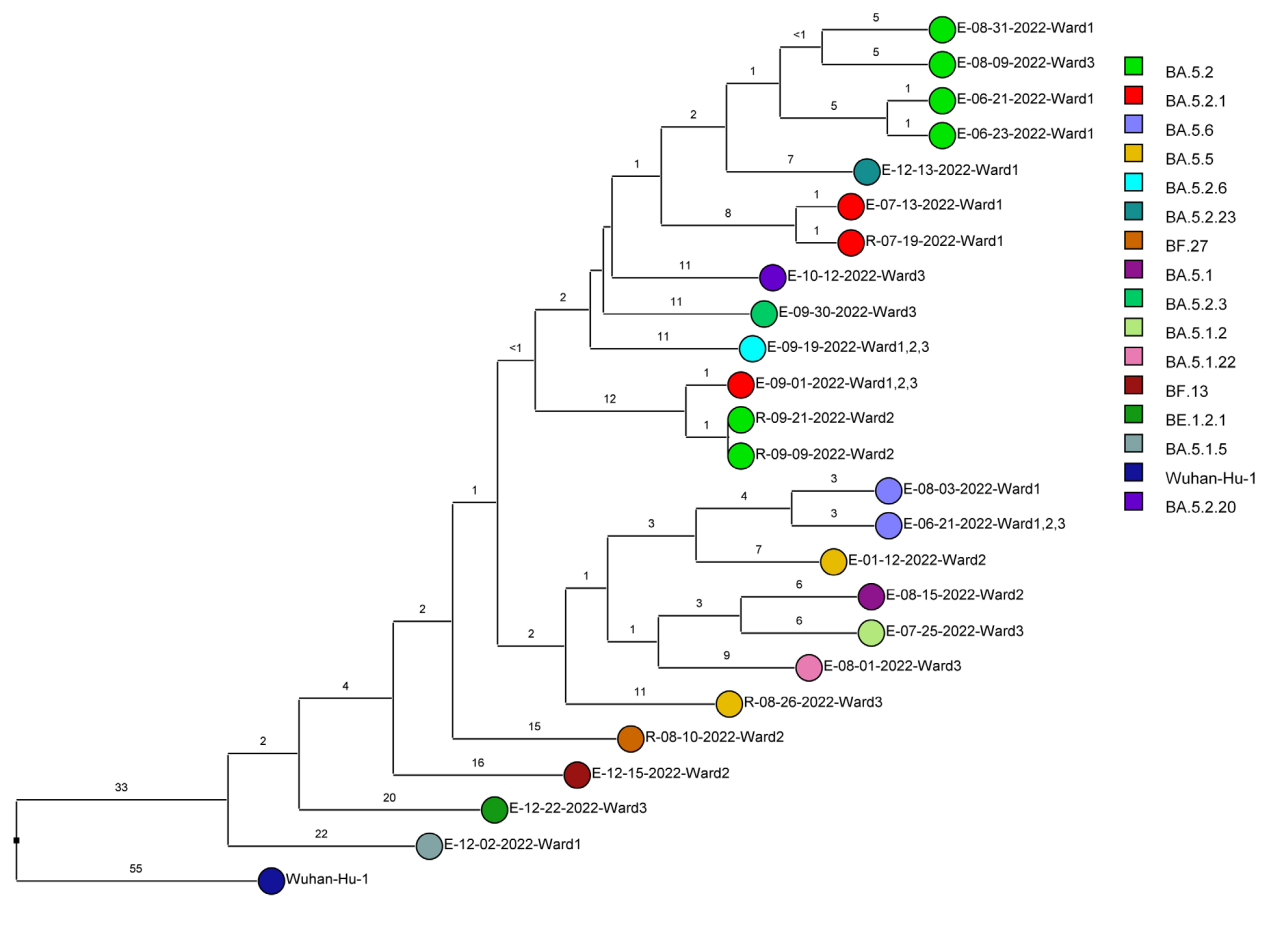
(Omicron BA.5, BE, BF [n=24])

**Figure 2G:**
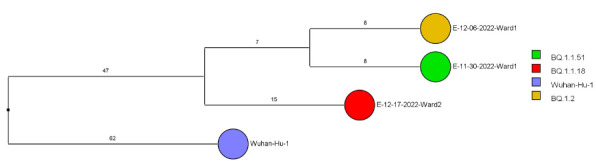
(Omicron BQ [n=3])

During the study period, there were 2 outbreaks with multiple transmission events that involved more than 1 floor of the community living center. For these outbreaks, sequencing analysis confirmed relatedness of many but not all the SARS-CoV-2 viruses from COVID-19 cases suspected to be linked based on contact tracing. Figure 3 provides a timeline for the 2 outbreaks showing only the SARS-CoV-2 samples that were genetically related; these samples are indicated in bold text in Figure 2A (labeled employees [E] 1-3 and residents [R] 1-2) and Figure 2C (labeled employees [E] 1-13 and residents [R] 1-2). In cluster A (Figure 3A), 2 employees on ward 2 with high-risk exposures to one another were diagnosed with COVID-19 on 12/2/20 and 12/3/20 and had related B.1.2 lineage sequences; 1 was symptomatic and the other was pre-symptomatic. Two residents on the ward that received care from 1 or both employees were subsequently diagnosed with COVID-19 and had related sequences; 1 was symptomatic and the other was asymptomatic. These residents had high risk contacts with one another. Sequencing analysis demonstrated that an employee on another ward (ward 1) was infected with a related B.1.2 lineage virus; this individual had a low-risk exposure to 1 of the infected employees on ward 2.

Figure 3.**Timeline for 2 of the coronavirus disease 2019 (COVID-19) clusters.** (A) Cluster of B.1.2 lineage infections; (B) Multiple ward cluster of BA.1.1 omicron lineage infections. Only the related (<2 single nucleotide polymorphism differences) severe acute respiratory syndrome coronavirus 2 (SARS-CoV-2) samples are shown. Vertical lines connecting rectangles indicate employees working on multiple wards. E, employee; R, resident.

**Figure 3A:**
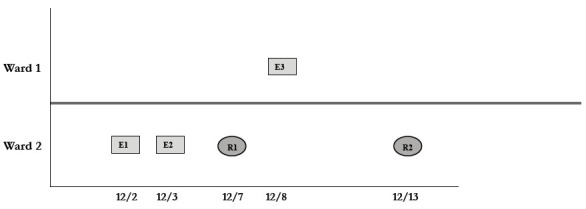
(B.1.2 lineage)

**Figure 3B:**
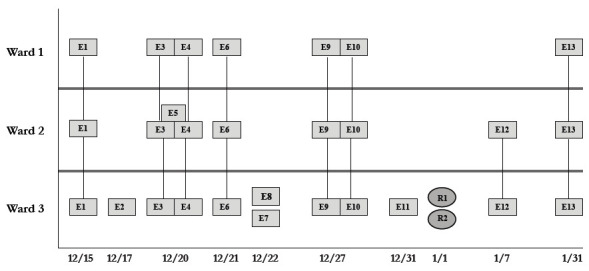
(BA.1.1 lineage)

Figure 3B provides a timeline for the second outbreak which was due to omicron BA.1.1 lineage SARS-CoV-2. For the 3 involved wards, contact tracing investigations at the time of the outbreak were restricted to individual wards, whereas sequencing demonstrated that all the wards had multiple infections with genetically related SARS-CoV-2. Of the 13 infected employees, 8 had jobs that required them to work on multiple wards (eg, physical therapy, pharmacy, environmental services, food service, and nursing assistant). Two residents on ward 3 were diagnosed with COVID-19 on 1/1/21; both had received care by 1 or more infected employees. The residents did not have contact with one another.

For multiple suspected transmission events based on contact tracing, sequencing demonstrated that the viruses were unrelated. For example, 2 suspected transmissions of delta variants (Figure 2B) based on contact tracing (resident 9/24/21 to employee 10/1/21 and employee 12/2/21 to employee 12/8/20) were demonstrated to be unrelated based on sequencing.

After the initial surge of omicron lineage infections in December and January 2021, multiple omicron lineages were detected (Figure 2D-2G). Based on contact tracing and sequencing, there were 2 episodes of transmission from employees to residents (employee 7/13/22 to resident 7/19/22 and employee 9/1/22 to resident 9/9/22) and a resident-to-resident transmission (resident 9/9/22 to resident 9/21/22). (Figure 2F). However, in most cases of suspected transmission based on contact tracing, the viruses were not genetically related based on sequencing. During this period, there were no restrictions on visitation except in outbreak situations. There were 2 episodes of suspected transmission from family members to residents, but samples were not available from family members to assess genetic relatedness.

### Carbon Dioxide Monitoring to Assess Adequacy of Ventilation

Carbon dioxide levels remained below 800 ppm in all sites tested. The peak carbon dioxide levels recorded over 10 hours in the nursing stations and physical therapy area were 468 and 548 ppm, respectively. The peak number of people in those areas were 7 and 16, respectively. In the conference room with 12 people present, carbon dioxide levels rose modestly as attendees entered the room peaking at 649 ppm. In resident rooms with 2 occupants, the peak carbon dioxide level was 610 ppm.

The expected ACH based on design specifications was 6 for resident rooms and 16 for the conference room. Specific ACH were not available for other sites in the facility, but engineering stated that 6 or more ACH would be expected for other occupied areas.

## DISCUSSION

During the 42-month study period, our infection control program conducted contact tracing investigations of multiple ward-level clusters of COVID-19. These investigations suggested that employees with COVID-19 were the major source of transmission to co-workers and residents. Sequencing analysis provided support for multiple transmission events between co-workers and from employees to residents. We identified one episode of likely transmission of SARS-CoV-2 from one resident to another, but there were no documented transmissions from residents to employees. Our findings are consistent with previous studies that identified nursing home employees as a primary source of transmission of SARS-CoV-2 to co-workers and residents [3, 6, 7, 9]. Healthcare employees in hospital and outpatient settings have also been implicated as a frequent source of transmission to co-workers [14].

Many healthcare personnel are concerned about the risk for acquisition of SARS-CoV-2 and other respiratory pathogens from patients or nursing home residents. During the study, there were several clusters in which transmission from nursing home residents to personnel was suspected based on contact tracing. However, in each of these clusters, sequencing analysis demonstrated that employees were infected with viruses that were unrelated to those infecting residents they cared for with prior infections. These findings highlight the value of sequencing analysis in confirming or refuting transmission in settings where there are multiple potential sources of exposure (ie, nursing home employees may acquire infection in the community or in the facility from co-workers or residents or visitors). Our results suggest that transmission from nursing home residents to employees may be uncommon in settings with adequate infection control measures in place (eg, employee masking for all interactions with residents, adequate ventilation). Several factors may have reduced the risk for resident to staff transmission in our facility. These include regular screening of residents for respiratory symptoms and fever, admission and weekly or twice-weekly RT-PCR or antigen testing, and transfer of infected symptomatic or asymptomatic residents to the hospital [8]. These control measures were instituted based on nationwide guidance from the Department of Veterans Affairs for community living centers [8].

One notable finding from our study was that outbreaks often involved multiple wards. Although contact tracing investigations focused on individual wards, sequencing analysis demonstrated that concurrent outbreaks on different wards were often due to genetically related variants of SARS-CoV-2. Many employees had jobs that required them to work on multiple wards. In some cases, such individuals were implicated as a potential vector for transmission between wards. Previous sequencing studies have demonstrated that transmission of other pathogens in nursing homes and hospitals often occurs in the absence of shared ward exposure [20–22]. For example, in our community living center, sequencing analysis demonstrated potential transmission of *Clostridioides difficile* from residents with asymptomatic carriage on 1 ward to residents on other wards who developed *C. difficile* infection [20].

Indoor areas with suboptimal ventilation pose a risk for airborne transmission of SARS-CoV-2 and other respiratory viruses [18]. In several nursing home outbreaks of COVID-19, inadequate ventilation has been identified as a potential contributing factor [10–13]. Based on carbon dioxide monitoring and a review of available information on air changes per hour, the ventilation in our community living center is sufficient to minimize the risk for airborne transmission [23, 24]. However, it should be noted that the air changes per hour in resident rooms in our facility (6 per hour) exceed the requirements for resident rooms in nursing homes (2 air changes per hour) [25]. The CDC recently recommended that all buildings upgrade ventilation systems to aim for at least 5 air changes each hour with minimum efficiency reporting value (MERV)-13 filters [23, 24]. However, the proportion of nursing homes that have improved ventilation systems to meet these recommendations is not known. Therefore, additional studies are needed to assess ventilation in other nursing homes.

Our study has several limitations. Only a subset of the cases had samples available for sequencing analysis, and many samples did not meet the stringent requirements for quality of sequencing. We did not sequence control samples from hospital employee and patient samples. Thus, we cannot be certain that some of the transmission events did not represent concurrent acquisition of related viruses widely circulating in the community or in the hospital. It is possible that we underestimated the sequence relatedness because we used stringent coverage metrics and Bionumerics 7.6 phylogenetic tree methodologies for concurrence. Finally, it is plausible that routes of transmission of SARS-CoV-2 might differ for nursing homes with less stringent infection control measures in place.

## CONCLUSION

Contact tracing and sequence analysis suggested that transmission of SARS-CoV-2 in the community living center predominantly involved transmission from employees to co-workers and residents. There is a need for improved measures to prevent transmission of SARS-CoV-2 by healthcare personnel in nursing homes.
